# In Situ Polyphosphate Nanoparticle Formation in Hybrid Poly(vinyl alcohol)/Karaya Gum Hydrogels: A Porous Scaffold Inducing Infiltration of Mesenchymal Stem Cells

**DOI:** 10.1002/advs.201801452

**Published:** 2018-11-13

**Authors:** Emad Tolba, Xiaohong Wang, Maximilian Ackermann, Meik Neufurth, Rafael Muñoz‐Espí, Heinz C. Schröder, Werner E. G. Müller

**Affiliations:** ^1^ ERC Advanced Investigator Grant Research Group at the Institute for Physiological Chemistry University Medical Center of the Johannes Gutenberg University Duesbergweg 6 55128 Mainz Germany; ^2^ Polymers and Pigments Department National Research Centre Dokki 12622 Giza Egypt; ^3^ Institute of Functional and Clinical Anatomy University Medical Center of the Johannes Gutenberg University Johann Joachim Becher Weg 13 55099 Mainz Germany; ^4^ Institute of Materials Science (ICMUV) Universitat de València C/Catedràtic José Beltrán 2 46980 Paterna València Spain

**Keywords:** coacervate, human mesenchymal stem cells, inorganic polyphosphate, karaya gum, nanoparticles

## Abstract

The preparation and characterization of a porous hybrid cryogel based on the two organic polymers, poly(vinyl alcohol) (PVA) and karaya gum (KG), into which polyphosphate (polyP) nanoparticles have been incorporated, are described. The PVA/KG cryogel is prepared by intermolecular cross‐linking of PVA via freeze‐thawing and Ca^2+^‐mediated ionic gelation of KG to form stable salt bridges. The incorporation of polyP as amorphous nanoparticles with Ca^2+^ ions (Ca‐polyP‐NP) is achieved using an in situ approach. The polyP constituent does not significantly affect the viscoelastic properties of the PVA/KG cryogel that are comparable to natural soft tissue. The exposure of the Ca‐polyP‐NP within the cryogel to medium/serum allows the formation of a biologically active polyP coacervate/protein matrix that stimulates the growth of human mesenchymal stem cells in vitro and provides the cells a suitable matrix for infiltration superior to the polyP‐free cryogel. In vivo biocompatibility studies in rats reveal that already two to four weeks after implantation into muscle, the implant regions containing the polyP‐KG/PVA material become replaced by initial granulation tissue, whereas the controls are free of any cells. It is proposed that the polyP‐KG/PVA cryogel has the potential to become a promising implant material for soft tissue engineering/repair.

## 1 Introduction

Advanced tissue engineering, as a branch of regenerative medicine, focuses on the replacement/regeneration of impaired/damaged tissues and organs to restore their biophysiological function within an organism. In the beginning of the last century, Alexis Carrel and Charles Lindbergh succeeded to keep entire organs alive outside of the body, using a perfusion machine (reviewed in: ref. 1). During this period, regenerative medicine was “cytocentric”, meaning that mainly the cells were assessed as the relevant players in biological complex systems (see: ref. 2). This concept changed with the conceptualization of the “OncoMouse or Harvard mouse” which introduced the technology of genetically modified laboratory animals to implant “relatively simple” body parts like vessels, bones or skin (ref. 3; reviewed in: ref. 4). The next phase started with the discovery that stem cells can initiate regeneration in complex tissue regions, which have lost this potency, through appropriate manipulation of those body parts. Those stem cells might re‐confer regeneration ability to quiescent cells (reviewed in ref. 5). It has been hoped that, e.g., mesenchymal stem cells (MSCs) can repair damaged tissue not only by recruiting other somatic cells but also by initiating the secretion of functional, bioactive molecules, like growth factors and matrix proteins. Finally, in a biomimetic approach scaffolds have been fabricated from natural polymers that are biodegradable, less immunogenic as compared to synthetic polymers, and provided with biomechanical properties matching those of the native components of the tissues with their extracellular matrix (ECM).^6^

At present, one major challenge in tissue engineering is the fabrication of a biomaterial as a scaffold that is not only biocompatible but also regeneratively active^7^, ^8^ through eliciting regeneration‐inducing stimuli by providing the metabolic energy that is required for the organization and reconstitution of the fibrillar and nonfibrillar ECM.^9^, ^10^ Such a scaffold, like thermoresponsive hydrogels,^11^, ^12^ should also meet the physical properties of the replaced tissue, besides of the (bio)chemical prerequisites.

Our group emphasized that it is inorganic polyphosphate (polyP) that acts as an extracellular storage for metabolic energy needed in the extracellular space.^9^, ^13^ Within the cells polyP is stored in vesicles, termed acidocalcisomes.^14^ These organelles contain polyP as a salt with inorganic divalent cations. In human platelets the chain lengths of polyP varies between 50 and 100 phosphate units.^15^ Applying a new technology, we succeeded to entrap polyP, in a biomimetic way, into amorphous nanoparticles (Ca‐polyP‐NP) formed by the Ca^2+^ salt of this polymer,^16^ like those present in acidocalcisomes; similar particles have also been synthesized with other divalent counterions of the polyP.^17^ These polyP nanoparticles have been shown to be morphogenetically active in vitro, both as nanoparticles and microspheres,^18^ as well as in vivo.^19^ Recently we showed that Ca‐polyP‐NP undergo coacervation after contact with proteins/peptides that reduce the zeta potential of the particles.^20^ The polyP is prone to enzymatic hydrolysis with the alkaline phosphatase (ALP), the dominant intra‐ and extracellular enzyme that hydrolyzes the phosphoanhydride bonds which link the phosphate groups.^21^ As consequence, Gibbs free energy is released, which is at least partially stored in the energy‐rich phosphoanhydride bonds of adenosine 5′‐diphosphate (ADP)/adenosine 5′‐triphosphate (ATP).^22^ In turn, the extracellularly formed ADP undergoes enzymatic disproportionation to adenosine 5′‐monophosphate (AMP) and ATP, catalyzed by the adenylate kinase.^9^, ^13^

Most interesting is the fact that the amorphous polyP nanoparticles meet the prerequisites of being fully biocompatible and provided with comprehensive functionalities allowing tissue incorporation and initiating tissue regeneration (ref. 17; reviewed in: refs. 23, 24). They are suitable for fabrication of bioinspired scaffold materials in tissue engineering bone and cartilage.^7^ They are even suitable for application as an implant material that can be fabricated in a personalized way by using a molding procedure^25^ or 3D bioprinting.^26^ The polyP nanoparticles can also be fabricated in hybrid forms, e.g., with N,O‐carboxymethyl chitosan^27^ or with hyaluronic acid^8^; the nanoparticles retain their biological function. First steps toward the formulation of a morphogenetically active bioink, applicable for 3D‐bioprinting at lower cell concentrations, have been successfully passed.^28^

It is the aim of the present study to integrate the Ca‐polyP‐NP into a scaffold suitable for bone and cartilage regeneration. It is well established that hydrogels, being highly hydrated polymers, are promising replacement materials for impaired bone as well as damaged cartilage tissues, since they match the required mechanical performance (strength and toughness) of those tissues (reviewed in refs. 29, 30). Hence those nanoparticles were formed within a karaya gum (KG)/poly(vinyl alcohol) (PVA) hydrogel. PVA is a water‐soluble synthetic polymer with the cross‐formula [CH_2_CH(OH)]*_n_*,^31^ which has been used for hydrogels in wound care, or for textiles or coatings. Since PVA readily forms hydrogen bonds in a symmetrical and regular arrangement, the polymer shows excellent filmability, water solubility, emulsification, and adhesion properties.^32^ The properties of PVA can be further improved by addition of different polymers toward higher biocompatibility, porosity, or biodegradation.^33^ A further promising feature of PVA, besides of its biomechanical properties, is the possibility of including a repeated freeze−thaw method,^34^, ^35^ allowing physical cross‐linking of the PVA cryogel with other polymers and components. Among them is the natural polysaccharide KG^36^, ^37^ which can be grafted with PVA to a hydrogel suitable for adsorption of compounds out of the aqueous milieu or of fluids in general.^38^ KG is stabilized via ionic cross‐linking with Ca^2+^.

In this study, we show that Ca‐polyP‐NP can be formed in situ within a KG:PVA copolymer cryogel where they retain their biological and morphogenetic properties. Like in the free state, the nanoparticles within the cryogel undergo coacervation after transfer of the material into a protein/peptide‐containing medium and by that become biochemically active.^20^, ^39^ The material is a dynamic hydrogel with pronounced viscoelastic properties. Incubation of the KG:PVA material with MSCs revealed that the cells respond with an increased growth potential and even display a higher propensity to infiltrate into the cryogel if the KG‐PVA gel, containing polyP in the form of Ca‐polyP nanoparticles, is used. Biocompatibility studies in vivo, performed by implanting the material into the muscle of rats, revealed that the polyP‐containing KG‐PVA material is superior to the polyP‐lacking cryogel. The data show that polyP nanoparticle formation can be performed in situ in a cryogel, qualifying this material as a potential new implant material offering new possibilities for soft tissue repair.

## 2 Results

### 2.1 Preparation and Characterization of the Karaya Gum/Poly(vinyl alcohol) Hydrogel

**Figure 1:**
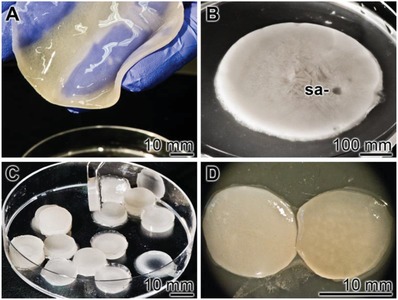
Fabrication of KG/PVA‐cryogel samples. A) After mixing of KG with PVA the viscous solution was poured into a Petri dish. B) During the subsequent three freezing‐thawing cycles the layer shrunk to ≈75%. From those layers samples (sa) were drilled out which C) were submersed into a 5% CaCl_2_ solution, allowing ionic gelation of the KG. The polyP was added as Na‐polyP prior to the ionic gelation. D) A cryogel slice without (left) and with polyP (right) is shown.

The hydrogel was prepared from KG and PVA after separate dissolution of these components in distilled water, as described under in the Experimental Section. After mixing and degassing, the viscous solution was poured into a Petri dish (**Figure** **1**A) and subsequently subjected to a freezing‐thawing cycle to obtain a 3D structured KG/PVA hydrogel and allowing physical cross‐linking of PVA. During this process the hydrogel layer shrunk by ≈25% (Figure 1B). From those 0.2 to 3 mm thick layers smaller units were excised/punched out with a scalpel or a trephine, which were submersed into a 5% CaCl_2_ solution (Figure 1C) to induce ionic cross‐linking of the KG (ionic gelation). Three different weight ratios for KG and PVA have been chosen; 1:2, 1:1, and 2:1 (KG:PVA). Accordingly, the cryogels were termed “KG1/PVA‐cryogel”, “KG2/PVA‐cryogel”, and “KG3/PVA‐cryogel”. The polyP was added to the material after cryogel formation and prior to ionic gelation initiated with 5% CaCl_2_ (Figure 1D). The samples with polyP are termed “KG1/PVA/polyP:NP‐cryogel”, “KG2/PVA/polyP:NP‐cryogel”, and “KG3/PVA/polyP:NP‐cryogel”.

Incubation of the lyophilized KG/PVA cryogel, either in the absence of polyP (“KG2/PVA‐cryogel”) or containing this polymer (“KG2/PVA/polyP:NP‐cryogel”), in phosphate buffered saline (PBS) did not result in a significant change (*p* < 0.05) of the weight of the dried scaffold after a 10 days' period.

*Mass Swelling Ratio*: The cryogel shows an extensive property to take up aqueous solution. The uptake is dependent on the concentration ratio between KG and PVA. Increasing the KG portion from 1:2 (“KG1/PVA‐cryogel”) to 1:1 (“KG2/PVA‐cryogel”) and to 2:1 (“KG3/PVA‐cryogel”) results in an intensified PBS uptake from 3.40 ± 0.08 to 4.29 ± 0.13 and finally to 5.26 ± 0.19 during a 1 h incubation period (**Table** **1**). A prolongation to 24 h does not change the swelling ratio significantly. Supplementation of the cryogel with polyP causes a significant increase in the swelling ratio for “KG1/PVA/polyP:NP‐cryogel” to 3.82 ± 0.15, and for “KG2/PVA/polyP:NP‐cryogel” and “KG3/PVA/polyP:NP‐cryogel” to 4.72 ± 0.17 and 5.69 ± 0.29, respectively. We attribute this increase to the superior hydrophilic properties of KG and to an increased Ca‐polyP nanoparticle formation,^20^, ^39^ paralleled with a decrease in hydrogen‐bonding between the polymer chains allowing a stronger uptake of water within the hydrogel network.

**Table 1:** Swelling ratios of the different cryogel compositions after swelling in PBS. The means ± SD have been calculated from 10 independent experiments

Sample designation	Mass swelling ratio (after 1 h)	Mass swelling ratio (after 24 h)
“KG1/PVA‐cryogel”	3.40 ± 0.08	3.65 ± 0.60
“KG2/PVA‐cryogel”	4.29 ± 0.13	4.36 ± 0.19
“KG3/PVA‐cryogel”	5.26 ± 0.19	5.30 ± 0.30
“KG1/PVA/polyP:NP‐cryogel”	3.82 ± 0.15	4.02 ± 0.90
“KG2/PVA/polyP:NP‐cryogel”	4.72 ± 0.17	4.98 ± 0.18
“KG3/PVA/polyP:NP‐cryogel”	5.69 ± 0.29	5.81 ± 0.23

**Figure 2:**
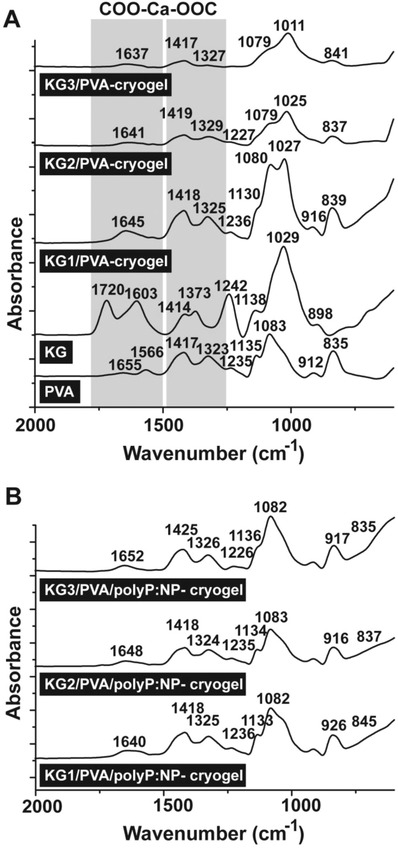
Characterization of the cryogels by FTIR. The signals between the wavenumbers 2000 and 600 cm^−1^ have been recorded; those that are marked are discussed in the text. A) Cryogels with the components PVA and KG. In addition, the spectra for “KG1/PVA‐cryogel”, “KG2/PVA‐cryogel”, and “KG3/PVA‐cryogel” are shown. B) The spectra of the polyP‐containing cryogels “KG1/PVA/polyP:NP‐cryogel”, “KG2/PVA/polyP:NP‐cryogel”, and “KG3/PVA/polyP:NP‐cryogel”. The shift of those bands which are caused by the interaction between the KG‐carboxylic groups and Ca^2+^ are boxed and shaded (COO‐Ca‐OOC) in (A).

*Fourier Transform Infrared Spectroscopy (FTIR) Analysis*: All the samples show a broad band in the region 3550 and 3200 cm^−1^ reflecting the stretching vibration of ν(O‐H) that originates from the intra/intermolecular hydrogen bonds of the PVA and KG chains and two bands around the region 3000 and 2800 cm^−1^ which refer to the stretching of ν(C—H) from the alkyl groups of the PVA and KG backbones (these data are not shown). A comparative FTIR spectral analysis of the KG/PVA‐cryogels and the KG/PVA/polyP:NP‐cryogels with their individual components KG and PVA in the region between 2000 and 600 cm^−1^ is given in **Figure** **2**. For PVA, the weak bands between 1670 and 1550 cm^−1^ represent the stretching vibrations of the remaining acetyl groups. The peaks located at 1417, 1323, 1235 cm^−1^ most likely reflect the bending vibration (δ) of (CH_2_), wagging vibration (ψ)‐(CH_2_) and ψ for C—H, respectively. The stretching vibrations for ν(C—O), ν(C—O—C), ν(C—C), and ψ(CH_2_) within the backbone of PVA polymer chains are observed at 1135, 1083, 912, and 835 cm^−1^, respectively.^40^ The spectrum for KG shows the vibration bands at 1720 and 1242 cm^−1^ which are due to the stretching ν(C=O) and ν(C—O) from the acetate group, vibrations of the carboxylic group ν(COO^−^) at 1603 and 1414 cm^−1^, the ν(C—H) vibration of methyl coming from the acetyl group at 1373 cm^−1^ and the ν(C—O) stretching and group vibration of the sugar rings at 1180–940 cm^−1^ (Figure 2A).^41^

The FTIR spectra of the different “KG/PVA‐cryogels” show the major absorption peaks that are found also in KG and PVA, with some structural modifications.^42^ In all cryogels new absorption bands within the regions 1700–1600 and 1400–1300 cm^−1^ are observed, which are due to the stretching vibration of the carbonyl groups (C=O), the acetyl as well as the carboxylic groups. These shifts can be attributed to the cross‐linking of the KG with its carboxylic groups to the Ca^2+^ ions during ionic gelation and additionally also the formation of hydrogen bonds between the hydroxyl groups of KG and the PVA. It is notable that the intensities of the absorption peaks originating from the acetyl group decreased, reflecting a reduction of the free acetate groups within the KG occurring during the preparation (Figure 2A).

With respect to the polyP‐containing cryogels, the absorption signal for the carboxylic group was also shifted, i.e., the carboxylic band at 1645 of “KG1/PVA‐cryogel” was shifted to 1640 cm^−1^ for “KG1/PVA/polyP:NP‐cryogel” (Figure 2B). Furthermore, the “KG1/PVA‐cryogel” bands close to 1325 cm^−1^ were decreased with addition of polyP (“KG1/PVA/polyP:NP‐cryogel”), as well as the 1080 and 1027 cm^−1^ were shifted to 1133 and 1082 cm^−1^ for “KG1/PVA/polyP:NP‐cryogel”. These modifications are also seen in the spectra for “KG2/PVA/polyP:NP‐cryogel” and “KG3/PVA/polyP:NP‐cryogel” which could be attributed to the incorporation of polyP within the KG/PVA‐cryogel and reflect the ionic interactions between the Ca^2+^ ions, the carboxylic group of the KG and polyP.^16^ Taken together, the above results indicate the in situ formation of Ca‐polyP nanoparticles within the KG/PVA cryogel (Figure 2B).

**Figure 3:**
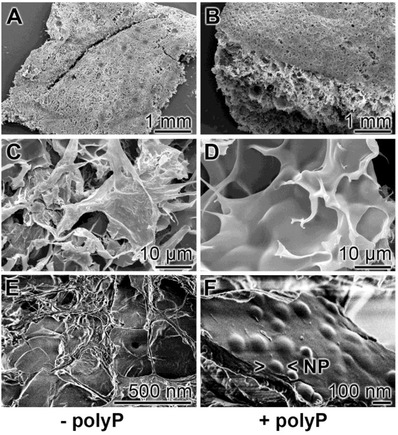
Morphology of the KG/PVA cryogel. A,C,F) The polyP‐free “KG2/PVA‐cryogel” sample and B,D,F) the polyP containing “KG2/PVA/polyP:NP‐cryogel” sample were analyzed; SEM. A–D) At lower magnification the cryogels comprises a meshwork, interspersed with extensive porous/channel‐traversed lacunas and openings. Using high resolution SEM microscopy the plain surfaces of the polyP‐lacking “KG2/PVA‐cryogel” samples are devoid of any nanoparticles (E), while those formed in the “KG2/PVA/polyP:NP‐cryogel” are abundantly interspersed with nanoparticles (NP) (F). The corresponding partial images are in the same magnification.

*Scanning Electron Microscope (SEM) Morphology*: The samples were analyzed by SEM (**Figure** **3**). Applying the low‐resolution SEM apparatus for lower magnifications the dry samples appear as an extensive and widespread porous/channel‐traversed material (Figure 3A–D). The dimensions of the channels reach up to 50 µm, irrespectively if the polyP lacking “KG2/PVA‐cryogel” (Figure 3A,C) or the polyP supplemented “KG2/PVA/polyP:NP‐cryogel” is analyzed (Figure 3B,D). Applying the ImageJ analysis software, the pore size of the freeze‐dried “KG2/PVA‐cryogel” was determined with 28 ± 8 µm and for the “KG2/PVA/polyP:NP‐cryogel” with 34 ± 9 µm.

At a high‐resolution SEM inspection it becomes overt that the plain surfaces within the cryogel in the samples lacking polyP do not show any nanoparticles (Figure 3E), while they are present in a high density in the polyP‐containing “KG2/PVA/polyP:NP‐cryogel” sample (Figure 3F). The nanoparticles are spherical and comprise a diameter of 100–150 nm.

**Figure 4:**
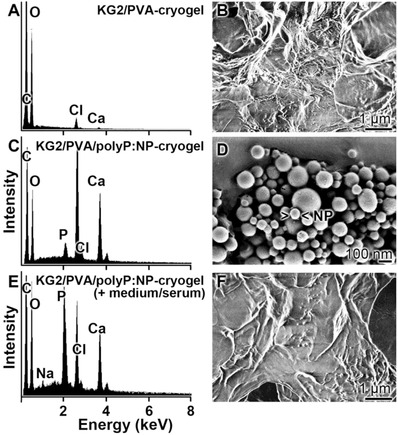
A,C,E) EDX spectra of the cryogels, prepared A,B) in the absence [“KG2/PVA‐cryogel”] or C,D) in the presence of polyP [“KG2/PVA/polyP:NP‐cryogel”], together with the B,D,F) corresponding SEM images. In (E) and (F), the cryogel was incubated for 1 h at 37 °C in DMEM medium with 10% FCS, prior to analysis. It is evident that the signals for P and Ca are present in the “KG2/PVA/polyP:NP‐cryogel”, while they are not visible in the “KG2/PVA‐cryogel”.

*Energy Dispersive X‐Ray (EDX) Analysis/Phosphate Content*: The polyP‐free cryogel “KG2/PVA‐cryogel” (**Figure** **4**B) shows no signals for phosphorus or calcium, if analyzed by EDX spectroscopy (Figure 4A). Only two peaks for carbon and oxygen are recorded. In contrast, inspection of the “KG2/PVA/polyP:NP‐cryogel” (Figure 4D) revealed besides of the peaks for C and O, within the electromagnetic emission spectrum, strong signals for Ca and P, comprising 4.0 wt% for Ca and 3.6 wt% for P (semi‐quantitative estimation) (Figure 4C). If these signals would originate from the complex between Ca^2+^ and phosphorus, a ratio of 0.66 wt% would be expected. However, at present the distribution of Ca^2+^ and PO_3_^−^ within the nanoparticles is not known; we expect a higher ratio than 0.66 wt%, because the particles are only formed in the presence of a surplus of Ca^2+^.^16^ In addition, KG also comprises some Ca^2+^ as a counterion to galacturonic acid.^43^

The presence of Cl in the EDX spectrum cannot be attributed to a potential NaCl contamination, since the signal for Na is lacking in the spectrum. If the KG sample “KG2/PVA/polyP:NP‐cryogel”, after incubation (1 h at 37 °C) in Dulbecco's modified Eagle medium (DMEM) with 10% FCS (Figure 4F), is analyzed, almost the same content for P and Ca is found with 3.8 wt% for Ca and 3.3 wt% for P (Figure 4C).

**Figure 5:**
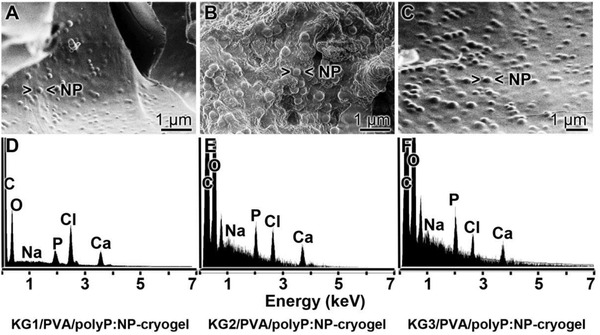
Morphology and composition of the KG/PVA cryogel, containing in situ formed Ca‐polyP nanoparticles, in dependence on the content of KG. Samples of KG:PVA were prepared in a ratio from 1:2 to 1:1 and finally to 2:1. The gels were supplemented with polyP and exposed to Ca^2+^ in order to initiate in situ nanoparticle formation. The gels are termed A) “KG1/PVA/polyP:NP‐cryogel”, B) “KG2/PVA/polyP:NP‐cryogel” [sub‐surface region showing the porous morphology], and C) “KG3/PVA/polyP:NP‐cryogel”. D–F) In a separate series of experiments, the polyP‐containing samples were analyzed by EDX. The corresponding spectra are given.

The content of polyP within the cryogel is dependent on its composition with respect to KG and PVA (**Figure** **5**). At all concentration ratios tested, 1:2 (KG:PVA) via 1:1 to 2:1, and using those samples as a matrix for in situ nanoparticle formation starting with Na‐polyP, the particles can be visualized by SEM (Figure 5A–C). Semi‐quantitative analysis of the cryogel specimens by EDX revealed that “KG1/PVA/polyP:NP‐cryogel” contains 2.1 wt% for Ca and 1.3 wt% for P (Figure 5D), reflecting a phosphate content of ≈3.3 wt%. For “KG2/PVA/polyP:NP‐cryogel” (Figure 5B), Ca–P wt% of 4.1 and 3.6 (phosphate content of ≈9.1 wt%) (Figure 5E) are determined, and for the “KG3/PVA/polyP:NP‐cryogel” (Figure 5C) 4.5 wt% for Ca and 3.7 wt% for P (phosphate of ≈9.5 wt%) (Figure 5F).

**Figure 6:**
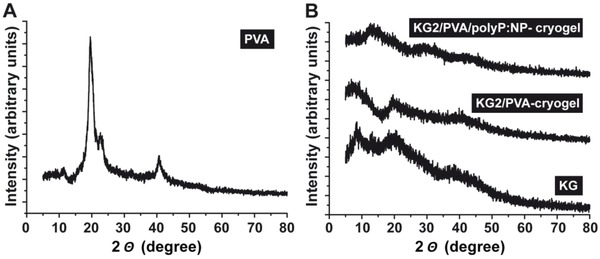
Characterization of A) PVA and B) KG starting materials, as well as of the polyP‐lacking cryogel, “KG2/PVA‐cryogel”, and of the polyP‐containing material “KG2/PVA/polyP:NP‐cryogel” by X‐ray diffraction (XRD). While the PVA pattern reflects crystallinity, the KG‐containing cryogels are amorphous.

*X‐Ray Diffraction* (*XRD) Patterns*: The XRD patterns of the pure PVA and KG powders as well as the fabricated “KG2/PVA‐cryogel” and the “KG2/PVA/polyP:NP‐cryogel” were recorded (**Figure** **6**). As shown, the pure PVA material exhibits a sharp intense peak at 2θ of 19.7° and adjacent weak intense peaks at 2θ of 11.6°, 22.7°, and 40.8° (Figure 6A). This XRD pattern reflects the semi‐crystalline nature of PVA, originating from the strong intermolecular interactions within the PVA chains through hydrogen bonds.^44^ In contrast, KG, as well as the “KG2/PVA‐cryogel” and the “KG2/PVA/polyP:NP‐cryogel” patterns do not show any sharp diffraction peak, indicating a highly disordered structure and amorphous nature of the KG used (Figure 6B). It is notable that within the XRD patterns of “KG2/PVA/polyP:NP‐cryogel” around 2θ of 15°, 23°, and 30° signals appeared that can be attributed to the presence of the Ca‐polyP NP which were formed in situ within the cryogel matrix. This result again supports the amorphous phase of the in situ synthesized Ca‐polyP NP. Taken together, the data show that KG changes the ordered structure and the intense packing of PVA.

### 2.2 Transformation of the Nanoparticles in the Cryogel to Coacervate

**Figure 7:**
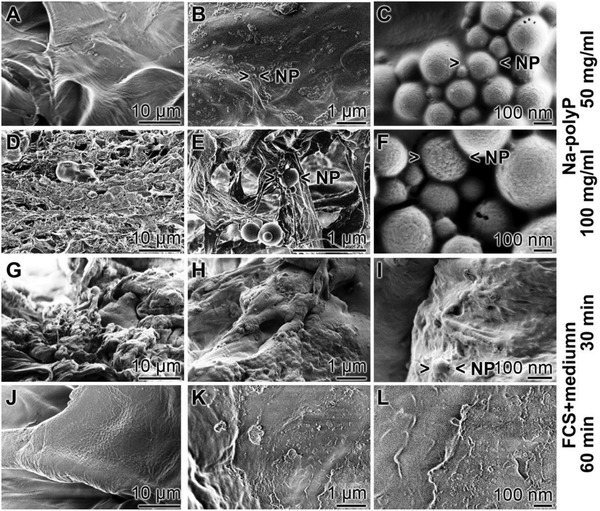
Transformation of the nanoparticles within the polyP‐containing “KG2/PVA/polyP:NP‐cryogel”; SEM analysis. KG/PVA was exposed either to A–C) 50 mg mL^−1^ Na‐polyP (surface view) or to D–F) 100 mg mL^−1^ Na‐polyP (showing the internal morphology) after the addition of CaCl_2_ solution (as described under “Experimental Section”) to induce in situ nanoparticle formation; SEM analysis. The morphology of the cryogel containing the polyP nanoparticles (> <NP) was likewise inspected by SEM in the dried state, G–I) 30 min or J–L) 60 min after incubation of the “KG2/PVA/polyP:NP‐cryogel” at 37 °C in medium/FCS. After the 60 min incubation period no polyP particles can be visualized.

In a recent study it was found that the polyP nanoparticles undergo coacervation after exposure to peptides/proteins.^20^ In the absence of peptides the nanoparticles in the dried “KG2/PVA/polyP:NP‐cryogel” remain permanently present (**Figure** **7**A–F). The sizes of the particles, formed in situ after exposure of the KG/PVA cryogel to 50 mg mL^−1^ Na‐polyP varies around 173 ± 70 nm (30 determinations) (Figure 7A–C (surface view of the hydrogel)). The porous structure of the cryogel is obvious. In contrast, if the hydrogel is exposed to 100 mg mL^−1^ Na‐polyP the formed particles are larger with 210 ± 85 nm (Figure 7D–F (the inner porous morphology is visible)).

Also, after immersing in PBS the particles are persistently existent for at least 2 weeks at room temperature (not shown here). In contrast, if the polyP‐containing cryogel is incubated for 30 min (Figure 7G–I) or 60 min at 37 °C in medium/FCS (Figure 7J–L) only a few nanoparticles are scattered within the hydrogel, especially at the shorter incubation period. However, this cryogel contains still phosphorus and calcium at the same proportion like in the nonincubated sample (previous paragraph). This finding strongly suggests that the polyP in the cryogel exists in the coacervate state.

### 2.3 Mechanical Properties

**Figure 8:**
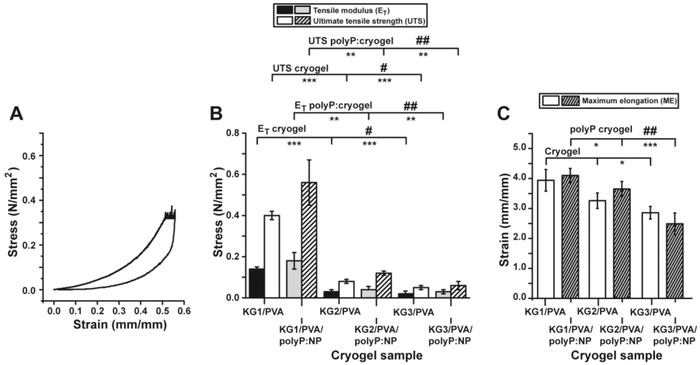
Mechanical properties of the fabricated material. A) One exemplary stress/strain curve, the relationship for the “KG2/PVA‐cryogel”, is shown. The nonlinear ascending branch is followed by a distinct plateau which reflects the creep phase. The descending branch shows a delayed recovery. The prominent hysteresis loop is indicative for a viscoelastic material. B) Mechanical properties of the materials (absence or presence of polyP) with respect to the tensile modulus and the ultimate tensile strength. C) Maximum elongation characteristics. The significance was calculated for both the *E*_T_ values (absence and presence of polyP) and for the UTS values (minus and plus polyP). The values for the polyP‐free “KG2/PVA‐cryogel” and “KG3/PVA‐cryogel” have been correlated either to the “KG1/PVA‐cryogel” (significance levels are indicated by asterisks [*]) or among each other (“KG2/PVA‐cryogel” and “KG3/PVA‐cryogel”; indicated by hashes [#]). The correlation of the polyP‐containing cryogels has been performed accordingly. The mean values ± SD with their significance levels are given (*[#], *p* < 0.05; **[##], *p* < 0.01; ***, *p* < 0.001) versus control, *n* = 10.

**Figure 9:**
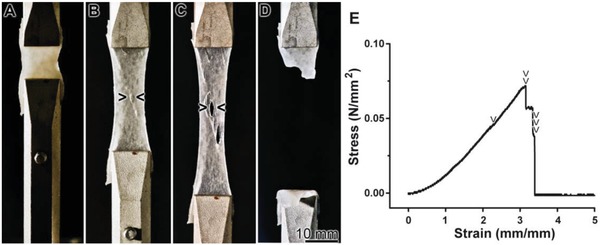
Tensile strength and elongation properties, using the “KG2/PVA/polyP:NP‐cryogel” material as an example. A) At the start of the experiment, the material is entirely homogeneous. B,C) With the progression of the experiment and the increase of the tensile force the material shows local ruptures (> <). D) After a 3.4‐fold stretching the material breaks. E) The corresponding tensile test, curve of the stress/strain relationship, with the “KG2/PVA/polyP:NP‐cryogel” as the example. Stress and strain increased during the continuous stretching of the specimen. The discontinuous transitions within the curve are indicative for proceeding defects in the integrity of the material. At first, small ruptures appear at 300% strain (2 mm mm^−1^) (one arrowhead) (referring to B); then, after increasing the strain to 410% larger fissures of the material appear (two arrowheads; corresponding to C). Finally at ≈440% strain the material fails and the stress level returns to zero (three arrowheads; corresponding to D).

The mechanical properties of the specimens without (“KG1‐3/PVA‐cryogel”) or with the polyP (“KG1‐3/PVA/polyP:NP‐cryogel”) were assessed by force‐displacement‐time measurements under longitudinal oriented compressive or tensile loading conditions. The resulting stress–strain curves clearly show a soft‐tissue like nonlinear viscoelastic behavior of the material^45^; a representative graph is given (**Figure** **8**A). The graph shows a typical viscoelastic stress strain behavior with a distinct hysteresis loop. The ascending branch is nonlinear and followed by a small plateau which reflects the time‐dependent creep phase. The descending branch shows a short, delayed but complete recovery of the material. A comparative analysis between the different polyP‐free and polyP‐containing KG/PVA materials shows that in both series the materials with the higher KG content, like in “KG3/PVA‐cryogel” and “KG3/PVA/polyP:NP‐cryogel”, show a decrease in the mechanical resistance of the respective specimens as seen from the data as follows. “KG1/PVA‐cryogel” shows a compressive modulus/stiffness (*E*_C_) of 42.44 ± 0.01 kPa a value that decreases significantly to 11.25 ± 01 kPa for “KG2/PVA‐cryogel”; the corresponding value for “KG3/PVA‐cryogel” is with 13.39 ± 01 kPa again significantly lower than the one for “KG2/PVA‐cryogel”. The tensile tests, which were independently performed (Figure 8B,C; **Figure** **9**), confirmed these changes. The values, reflecting the material properties, and correlating with the ultimate tensile strength (UTS) were found to be for the “KG1/PVA‐cryogel” 399.37 ± 14.74 kPa, while for the “KG2/PVA‐cryogel” a sharp decrease is determined with 81.32 ± 10.15 kPa and for “KG3/PVA‐cryogel” a further decrease to 45.74 ± 7.21 kPa is recorded (Figure 8B). Finally, the determinations revealed that “KG1/PVA‐cryogel”, which has the lowest KG to PVA ratio (1:2), shows the highest tensile stiffness (143.67 ± 5.51 kPa), while for “KG2/PVA‐cryogel” (32.35 ± 5.29 kPa) and “KG3/PVA‐cryogel” (19.57 ± 3.48 kPa) lower values are measured.

The assessment of the maximum elongation (ME) of the three materials shows only slight variations (Figure 8C). In general, the elongation behavior of “KG1/PVA‐cryogel” (3.94 ± 0.36 mm mm^−1^), “KG2/PVA‐cryogel” (3.26 ± 0.26 mm mm^−1^), and “KG3/PVA‐cryogel” (2.86 ± 0.21 mm mm^−1^) is almost identical. The values of “KG3/PVA‐cryogel” show significant differences, only if compared to “KG1/PVA‐cryogel”.

The addition of Na‐polyP to the materials did not result in significant changes of the mechanical properties. The values for the UTS for “KG1/PVA/polyP:NP‐cryogel” (564.75 ± 109.15 kPa), “KG2/PVA/polyP:NP‐cryogel” (118.25 ± 11.76 kPa), and “KG3/PVA/polyP:NP‐cryogel” (59.75± 23.06 kPa) are in a similar range like the corresponding materials without polyP. However, a substantial increase of the standard deviation for “KG1/PVA/polyP:NP‐cryogel” and “KG3/PVA/polyP:NP‐cryogel” is calculated (Figure 8B). This fact suggests to us a less homogenous behavior of the polyP enriched materials. Also for the *E*_T_ the determined values are in the same order like those determined in the material without polyP (“KG1/PVA/polyP:NP‐cryogel”: 178.53 ± 42.11 kPa; “KG2/PVA/polyP:NP‐cryogel”: 41.53 ± 2.65 kPa; “KG3/PVA/polyP:NP‐cryogel”: 28.25 ± 8.51 kPa). Again, the standard deviation for “KG1/PVA/polyP:NP‐cryogel” and “KG3/PVA/polyP:NP‐cryogel” increased. The ME of the materials with polyP addition shows no significant difference to the materials without polyP (“KG1/PVA/polyP:NP‐cryogel”: 4.10 ± 0.24 mm mm^−1^; “KG2/PVA/polyP:NP‐cryogel”: 3.65 ± 0.25 mm mm^−1^; “KG3/PVA/polyP:NP‐cryogel”: 2.49 ± 0.36 mm mm^−1^); the extent of the standard deviations remains unchanged.

The tensile properties of the KG‐PVA material are pictured in one image using the “KG2/PVA/polyP:NP‐cryogel” (Figure 9). At the beginning of the tensile tests the surface of the material is dense and closed; it appears to be homogeneous (Figure 9A). After increasing the strain to ≈300% small ruptures appear that increase in size (Figure 9B,C). After a 4.4‐fold total increase the samples tears (Figure 9D). The corresponding tensile stress‐strain curve is shown (Figure 9E) with the marked abrupt discontinuities of these phases.

In order to allow a correlation between those values for the fabricated cryogels with native tissue, one series of tensile experiments was performed with bovine muscle tissue. We oriented the muscle fibers perpendicular to the loading direction. The animal tissue gave a UTS value of 73.96 ± 11.93 kPa, an *E*_T_ of 84.74 ± 59.39 kPa and an ME value of 1.56 ± 0.46 mm mm^−1^. These values are close to the ones measured for the polyP‐free KG/PVA‐cryogel samples.

### 2.4 Cell Viability Studies

**Figure 10:**
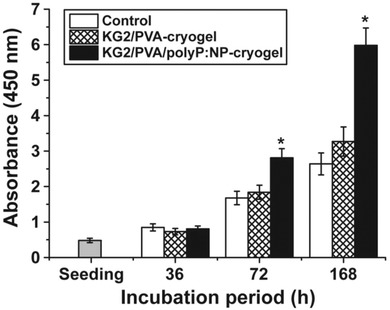
Viability studies using MSC. The cells were plated onto either hydrogel‐free wells (controls), or onto cryogel cushions lacking polyP (“KG2/PVA‐cryogel”) or the polyP‐containing material (“KG2/PVA/polyP:NP‐cryogel”). The numbers of viable cells were determined using the XTT assay (A_450_ values are given). Three incubation periods (36, 72, and 168 h) were selected. Ten parallel assays were performed and the mean values (±SD) are given. Significant correlations are determined within a given incubation period; they are marked (*, *p* < 0.001).

The MSCs were seeded into 6‐well plates which were either free of any cryogel (control) or contained the cryogel, either as polyP‐free cushion “KG2/PVA‐cryogel” or as polyP‐containing “KG2/PVA/polyP:NP‐cryogel”. The cell growth/viability was determined with the XTT assay system, as described under “Experimental Section” (**Figure** **10**). After an incubation period of 36 h the cell number increased to the same extent in all three series, as can be deduced from the increase in the absorbance from 0.48 ± 0.06 (at the seeding) to ≈0.8 absorbance units at 450 nm. After an incubation period of 72 h the cell concentration increased further to 1.68 ± 0.19 units (control) and 1.84 ± 0.21 (“KG2/PVA‐cryogel”), or to 2.81 ± 0.26 (“KG2/PVA/polyP:NP‐cryogel”), respectively. The significant increase in the growth rate, reflecting also the remarkable biocompatibility of the polyP‐containing hydrogel “KG2/PVA/polyP:NP‐cryogel” in comparison to the control or the “KG2/PVA‐cryogel” is even more pronounced after a total incubation period of 168 h.

### 2.5 Infiltration of the Stem Cells into the Cryogel

**Figure 11:**
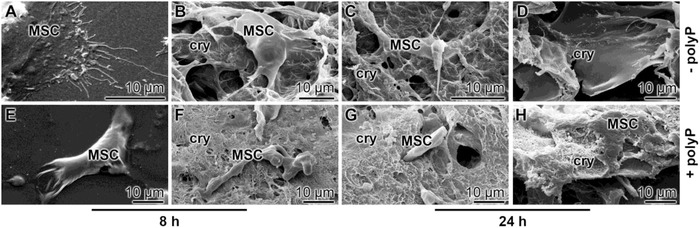
Infiltration of MSC into KG/PVA matrices. The cells were layered onto either A–D) the “KG2/PVA‐cryogel” or E–H) the “KG2/PVA/polyP:NP‐cryogel”. Then both series were incubated for 8 or 24 h, as indicated; subsequently, the samples were fixed, coated, and subjected to low‐resolution SEM. Primarily the MSCs attached onto the polyP‐containing cryogel (cry) were found to migrate into the channels of the gel, as in (H). A–C, E–G) Surface aspects and D,H) view of cross fractures.

As outlined under “Experimental Section” the polyP‐lacking and polyP‐containing cryogels were prepared onto the bottom of 6‐well plates. After 8 or 24 h the cells were fixed and inspected by SEM. In the series of cultivation onto “KG2/PVA‐cryogel” the cells adhere during the first 8 h to the cryogel matrix (**Figure** **11**A,B) and remain there also after 24 h (Figure 11C,D). In contrast, if the MSCs were plated onto the “KG2/PVA/polyP:NP‐cryogel” they appear to become coated with the hydrogel during the first 8 h (Figure 11E,F) or even infiltrate into the channel‐like system of the material (Figure 11G,H).

**Figure 12:**
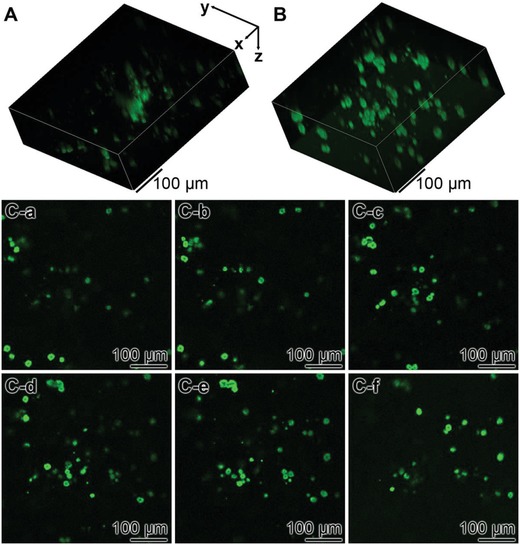
Analysis of the distribution of MSC within A) the “KG2/PVA‐cryogel” and B) the “KG2/PVA/polyP:NP‐cryogel” by cLSM. The cells were incubated for 8 h, then stained with Calcein AM and inspected. The compressed stacks are given in (A) and (B). In (C), individual slices from the poly‐free and the polyP‐containing cryogel (from the top to the bottom) are shown: C‐a–C‐c) aspects within the “KG2/PVA‐cryogel” and C‐d–C‐f) within “KG2/PVA/polyP:NP‐cryogel”. This series shows the increasing density of the cells within the polyP containing cryogel.

In order to verify that the cells are infiltrating into the cryogel the samples were inspected by confocal laser scanning microscopy (cLSM) (**Figure** **12**). The cells (20 × 10^3^ cells mL^−1^) were plated onto either the “KG2/PVA‐cryogel” or the “KG2/PVA/polyP:NP‐cryogel” and incubated for 8 h. Then the samples were stained and inspected by cLSM. The individual images were collected and the resulting 3D images were compiled. The computed 3D files showing the distribution of the cells distinctly show that only comparably few cells infiltrated into the “KG2/PVA‐cryogel” (Figure 12A), while the accumulation of the cells within the “KG2/PVA/polyP:NP‐cryogel” is more dense (Figure 12B). In order to document also the individual layers of the distribution of the cells within the polyP‐free (Figure 12C‐a–C‐c) and polyP‐containing gel (Figure 12C‐d–C‐f) individual images from the top to the bottom of the stack are given. Optical cell counts of defined areas within the cLSM stacks (100 µm × 100 µm × 100 µm) revealed an approximate number of 68 ± 28 cells, infiltrated within the “KG2/PVA/polyP:NP‐cryogel”, while only 18 ± 12 cells were counted within “KG2/PVA‐cryogel” samples.

### 2.6 In Vivo Biocompatibility Studies in Rats

**Figure 13:**
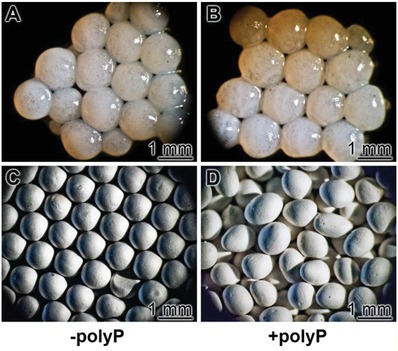
KG/PVA microspheres, used for the in vivo biocompatibility studies. The spheres contained as a scaffold KG and PVA and were prepared by an emulsion technology. A,C) The control, polyP‐free spheres (“KG/PVA:MS”); B,D) polyP‐containing microspheres “KG/PVA/polyP:MS”; A,B) light microscopic and C,D) SEM images.

The KG/PVA microspheres were prepared as described in the Experimental Section. An emulsion procedure was selected to obtain round microspheres of a diameter of 820 ± 70 µm (**Figure** **13**). The inner structure of the spheres is porous, interspersed with channels with dimensions ranging between 15 and 30 µm (not shown). No pronounced morphological differences between the polyP‐free “KG/PVA:MS” and the polyP‐supplemented spheres, “KG/PVA/polyP:MS”, were seen (Figure 13A,C and B,D). The polyP content of the “KG/PVA/polyP:MS” was determined to be 8.5 ± 0.7 wt%.

**Figure 14:**
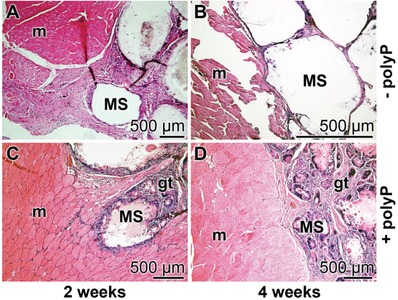
Implantation of the KG/PVA microspheres into back muscle regions of rats. A,B) Insertion of polyP‐free spheres (“KG/PVA:MS”); C,D) placing of polyP‐containing microspheres, “KG/PVA/polyP:MS”. Samples were taken after A,C) 2 weeks and B,D) 4 weeks, sliced, stained with hematoxylin and inspected. MS, microspheres; gt, granulation tissue‐assembly; m, muscle fasciae.

Samples of microspheres (20 mg) were implanted into the back of the rats, as described in the Experimental Section. After a healing period of 2 weeks or 4 weeks tissue samples were taken, sliced and stained with the hematoxylin. Eye inspection of the samples revealed that in none of the animals histopathological alterations evolved. Histological analyses of all sample sections of the two groups were performed. Typical images for the stained sample sections are shown (**Figure** **14**). It is striking that after 2 weeks the regions where the polyP‐free implanted microspheres, “KG/PVA:MS”, were placed contained “hollow” spaces which did not contain infiltrated cells (Figure 14A,B). In contrast, the regions into which the polyP‐supplemented spheres, “KG/PVA/polyP:MS”, were inserted were already partially replaced by cells forming an initial granulation tissue‐assembly (Figure 14C). An extension of the implant regeneration period to 4 weeks intensified the extent of tissue regeneration with “KG/PVA/polyP:MS”; granulated regions which contained initially the spheres were surrounding the muscle fasciae (Figure 14D). Those regions were not found in places which contained the polyP‐free samples (Figure 14B).

## 3 Discussion

Any kind of animal tissue is structured, based on the underlying fibrous network primarily due to the complex and organized collagen meshes. This characteristics is required for providing the tissue form, strength, and stability. Surely, the degree of complexity between different organs varies; among the highly hierarchically structured, fibrillar collagen‐based tissues are the cornea^46^ and the muscle.^47^ Focusing on muscle, this tissue is macroscopically composed of muscular fibers, built of myofibrils constructed from actin, myosin, titin, and other proteins that hold these fibrils together.^48^, ^49^ The muscles have viscoelastic properties since they comprise both viscous and elastic characteristics during the process of deformation. Especially if connected with the tendon, the muscles comprise a high degree of stress relaxation,^50^ optimizing this tissue for considerable stiffness and pronounced damping to the loading response.^51^

**Figure 15:**
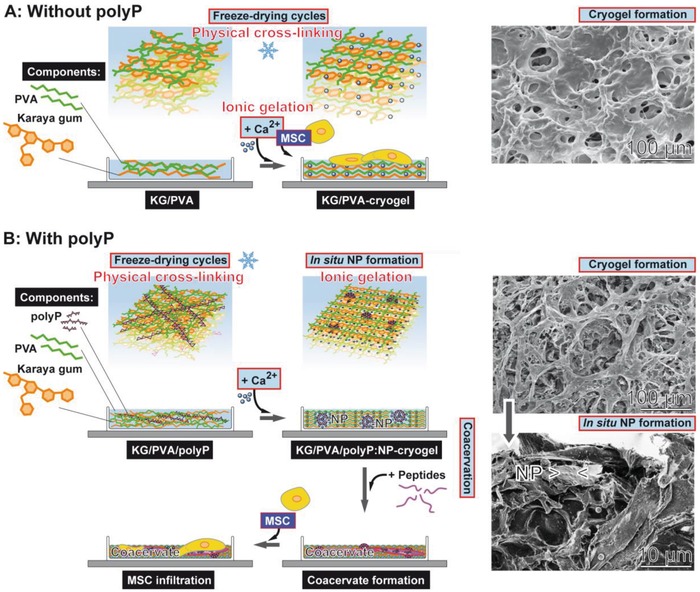
Formation of porous KG‐PVA hydrogel based on physically cross‐linked PVA (during the freezing‐thawing process) and on ionic gelation of KG (scheme flanked with SEM images on the right panel). A) Formation of the cryogel in the absence of polyP. The resulting material is porous (SEM insert). After further processing with Ca^2+^ a porous “KG/PVA‐cryogel” is formed that allows the MSC to attach. B) If Na‐polyP is added together with KG and PVA and subsequently processed by freeze‐thawing, the physical cross‐linkage of the PVA matrix occurs. The subsequent exposure to Ca^2+^ causes cross‐linkage of the KG under formation of the hybrid KG/PVA cryogel by ionic gelation (upper SEM image in panel B). In parallel 100–150 nm large Ca‐polyP nanoparticles (NP) are formed in situ that appear abundantly on the KG‐PVA matrices (lower SEM image in panel B). After lyophilization the material is stable and can be stored. If transferred to medium/serum the polyP‐containing cryogel, “KG/PVA/polyP:NP‐cryogel”, allows the nanoparticles to undergo coacervation; this matrix provides the MSC a suitable matrix to adhere and also to infiltrate into the cryogel.

Since the characteristics of most tissues is that they do not exhibit linear elasticity because of the presence of the locally and spatially distinct arrangement of the cells and their surrounding extracellular matrix, an artificial scaffold suitable for soft tissue repair is preferentially a hydrogel showing viscoelastic properties.^52^ Even more advanced would be hydrogels that are poro‐viscoelastic in order to facilitate vascularization.^53^ In the present study a hybrid viscoelastic hydrogel is introduced that is constructed from two polymer systems that are cross‐linked through two different interacting forces; first by PVA whose molecules are linked together via hydrogen bonds and second by KG which allows cross‐linking through their anionic sugar resides via Ca^2+^ (see Section 1). In a first step, the PVA/KG solution was subjected to freezing‐thawing cycles to introduce a porous channel system and to allow physical cross‐linking of the PVA chains to occur, and subsequently to Ca^2+^ that causes ionic gelation (Scheme in **Figure** **15**A). Both bonding types are noncovalent, allowing a considerable degree of flexibility and elasticity of the scaffold, which are prerequisites for cells to invade into the scaffold or to migrate onto this material. By alteration of the pH conditions in the vicinity of the cells or by peptides released by them and interacting with the ionic and hydrogen bonds the strength of the network is substantially affected.^20^ This property of the scaffold substantially controls the relaxation and retardation times of the MSCs, and by that also the viability and the differentiation capacity of those cells.^54^

The PVA/KG‐based cryogel comprises a considerable potential to take up water/PBS. For all cryogel species synthesized here the swelling ratio was larger than 3, qualifying the material as a suitable hydrogel for tissue engineering.^55^ Increasing the KG content increased the PBS uptake capacity by > 50%. The increase in the KG content also decreases the tear strength, without considerable effect on the elasticity of the cryogel. Likewise, the tensile modulus decreases allowing a lower elastic deformation of the material.

The mechanical properties of the PVA/KG‐based cryogel were compared with those of bovine muscle. Those studies revealed that the UTS value and, in parallel, also the *E*_T_ value are close to those measured for the KG/PVA‐cryogel samples, especially for “KG2/PVA‐cryogel” and “KG3/PVA‐cryogel”.^45^, ^56^

It is important to note that, in the XRD pattern, PVA shows sharp crystalline reflections, with a strong maxima at 2θ of 19.7° and adjacent weak intense peaks at 2θ of 11.6°, 22.7°, and 40.8°. This characteristic has been described earlier^57^ and is confirmed here. If PVA is added to KG, even under the different weight ratios selected, the amorphous phase of KG remains unchanged. This observation underscores the usefulness of the cryogel as a potential implant material in tissue engineering. This assessment bases on the observation disclosing that implants having an amorphous phase are, in general, more suitable than crystalline materials.^58^

The distinguished feature of the PVA/KG cryogel is its characteristics to allow an in situ synthesis/formation of nanoparticles from polyP and Ca^2+^ ions (Figure 15B). Usually hydrogels provide ideal environment to support cell adhesion and tissue formation, based on their bioinert nature.^59^ Additional functionalization of the materials can be introduced, e.g., by cell‐binding domains that serve as ligands for integrins and by that provide anchorage and triggering signals that direct cell function and the expression of the differentiated phenotypes.^60^ In the present study, a procedure is described that allows the in situ synthesis of nanoparticles of morphogenetically active polyP into the PVA/KG cryogel. Those Ca‐polyP particles have a size similar to the ones that have been previously synthesized in aqueous solution.^16^ The maximum content of polyP within the scaffold was around 9.5 wt%. This level is wanted since a much higher amount would open the risk of a local pH drop during the process of enzymatic hydrolysis by ALP.

Taken together, the fabricated PVA/KG cryogels show a tissue‐like nonlinear viscoelastic behavior with comparable maximum elongation values, as seen for muscle and cartilage.^45^, ^52^ The shift of the KG/PVA ratio to higher PVA contents leads to an increased tensile strength as well as a higher stiffness of the materials and vice versa. The addition of polyP does not result in a substantial alteration of the mechanical properties of the hybrid material.

It might be highlighted that both components of the scaffold described here, PVA and KG, are biocompatible and biodegradable.^38^, ^61^ PVA is more stiff and tough in comparison to KG.^62^, ^63^ This finding is corroborated by our studies revealing that an increased KG content of the cryogel decreased the Young's modulus.

In a recent study, we described that biomimetically fabricated Ca‐polyP nanoparticles are not degraded/metabolized unless they are transferred into the coacervate state.^20^ This transformation from the amorphous state as nanoparticles to the coacervate has been demonstrated to occur after transfer to a proteinaceous environment. Now, we show that this transformation to the biologically active polyP coacervate also proceeds with in situ formed Ca‐polyP nanoparticles. After exposure of the KG/PVA‐cryogel to a protein‐containing medium/serum the nanoparticles within the cryogel disappeared without changing the polyP content. These data are strong indications that the transition of the nanoparticles from the amorphous nanoparticulate state to the coacervate state also occurred in situ, implying that the bioavailability of the nanoparticles in the fabricated cryogel is not impaired.

As demonstrated^20^ the polyP‐containing cryogel, most of the studies have been performed with “KG2/PVA/polyP:NP‐cryogel”, provides an excellent matrix for MSCs to proliferate. The growth rate onto the polyP‐containing cryogel is higher than the one onto the polyP‐lacking cryogel. Intriguing is the observation that the cells cultivated onto the polyP‐containing hybrid cryogel show a high propensity to infiltrate in the matrix. This SEM observation was corroborated by cLSM analysis. In addition, previous studies have shown that in response to polyP the expression of the chemokine SDF‐1α is upregulated.^64^

In order to test the biocompatibility of the new hybrid material in vivo, the material was fabricated as microspheres using the emulsion technology,^65^ which were implanted into the muscle tissue. After a 4 week's regeneration phase the regions into which the polyP‐free microspheres were implanted were free of any cells. In contrast, the regions which were implanted with the polyP‐containing (better: in situ formed Ca‐polyP nanoparticles enriched) KG/PVA hydrogel were replaced by cells under formation of granulation tissue‐like assemblies. This finding highlights the beneficial component, polyP, in the microspheres as a morphogenetically active constituent.

## 4 Conclusion

The cryogel presented here is not only equipped with suitable, functionally adaptable tissue‐matching properties and space‐filling characteristics, but also with the potential to stimulate the growth of MSCs in vitro and—most importantly—accelerate the cell‐based repair/regeneration processes in vivo. The technology presented here, in which morphogenetically active nanoparticles are formed in situ, is a versatile method that might be applicable also for other hydrogel scaffold materials. Even though this study describes a one‐stage in situ synthesis procedure, an adaptation of the method to multistep processes appears to be straightforward. In ongoing studies the application of the in situ technology for the inclusion/supplementation of Ca‐polyP nanoparticles into i) microspheres, suitable to embed stem cells, ii) artificial, biomaterial‐based blood vessels, and iii) 3D printed implants is elaborated.

## 5 Experimental Section

*Materials*: Na‐polyphosphate (Na‐polyP) with an average chain length of 40 phosphate units was from Chemische Fabrik Budenheim (Budenheim; Germany).

*Preparation of the Porous Karaya Gum/Poly(vinyl alcohol) Hydrogel*: Ten grams of PVA (*M*_w_ 146 000–186 000; #363065 Sigma‐Aldrich, Taufkirchen; Germany) were dissolved in 100 mL of distilled water at 90 °C, while stirring for 3 h. In parallel, 5 g of KG (from sterculia tree; #G0503 Sigma) were dissolved in 100 mL of water at 90 °C for 3 h. Then, different volumes of the KG solution were added to a fixed amount of PVA and stirred for 1 h. In this study, three different weight ratios of KG:PVA were chosen, namely 1:2, 1:1, and 2:1. After mixing, the resulting KG/PVA viscous solutions were allowed to stand for 3 h to remove air bubbles. Subsequently, 20 mL of the respective KG/PVA solution were poured into a 12 cm Petri dish (plastic) and frozen down to −80 °C for 6 h. Afterward, the frozen dishes were thawed at room temperature for a period of 3 h. This freezing‐thawing cycle was applied three times to obtain a 3D structured KG/PVA hydrogel (based on physical cross‐linking of PVA chains). The obtained KG/PVA hydrogel was cut out by using a sharp biopsy punch of 10 mm inner diameter.

One batch was directly immersed into a calcium chloride solution (5%; CaCl_2_·2H_2_O) for 6 h under agitation to allow ionic cross‐linking of the KG (ionic gelation). The obtained KG/PVA hydrogel was washed three times with water to remove the nonreacted ions and freeze dried at −80 °C to obtain the KG/PVA hydrogel; the three samples were termed “KG1/PVA‐cryogel” (ratio between KG and PVA 1:2), “KG2/PVA‐cryogel” (ratio KG and PVA 1:1), and “KG3/PVA‐cryogel” (ratio KG and PVA 2:1). For the in situ synthesis of Ca‐polyP nanoparticles (NPs) within the KG/PVA matrix, the obtained hydrogels were immersed into Na‐polyP solution (1 g/20 mL distilled water) for 2 h, prior to the addition of 5% CaCl_2_ solution to allow the formation of Ca‐polyP NP within the KG/PVA hydrogel. In one series of experiments the concentration of Na‐polyP solution was increased to 2 g/20 mL water. The resulting samples were termed “KG1/PVA/polyP:NP‐cryogel”, “KG2/PVA/polyP:NP‐cryogel”, and “KG3/PVA/polyP:NP‐cryogel”. All the outlined samples were lyophilized prior to a further characterization.

The swelling ratio (*Q*_M_) was defined as the fractional increase of the cryogel in the weight of the hydrogel due to PBS (phosphate buffered saline) uptake and was calculated after incubation of the cryogel for different time periods at 37 °C according to the following equation; *Q*_M_ = mass of fully swollen gel/mass of dried hydrogel.^66^

*Stability of In Situ Formed Nanoparticles in the Cryogel*: Samples of 50 mg of “KG2/PVA‐cryogel” were exposed to 1 mL of PBS or to 1 mL of Dulbecco's modified Eagle medium (DMEM) with 10% FCS for a period of 60 min at 37 °C. Then the cryogel samples were washed and processed. In one additional series the samples, exposed to FCS+medium, were already inspected after 30 min by SEM.

*Fourier Transform Infrared Spectroscopy*: The analysis by FTIR was performed with an attenuated total reflectance‐FTIR spectroscope/Varian IR spectrometer (Agilent, Santa Clara; CA). The ground composite powder was analyzed.

*X‐Ray Diffraction*: The XRD patterns of dried powder samples were recorded with a D8 Advance A25 diffractometer (Bruker, Billerica, MA; USA) using monochromatic Cu‐Kα radiation.^67^

*EDX Analysis*: The experiments were performed with an EDAX Genesis EDX System attached to a scanning electron microscope (Nova 600 Nanolab, FEI, Eindhoven; The Netherlands). The analyses were operated at 10 kV with a collection time of 30–45 s. Areas of 10 µm^2^ were analyzed. In a first approximation, the signals corresponding to the different elements were used for a semi‐quantitative assessment.^68^

*Determination of the Mechanical Properties: Compression Testing*: In order to determine the bulk mechanical properties of the respective KG/PVA materials the samples were analyzed with the MultiTest 2.5‐xt force testing system (Mecmesin Ltd., Slinfold; UK), connected with the 100 N Load Cell unit. The experiments were performed with standardized cylindrical scaffold samples which were punched out from the sample material using a stainless steel biopsy trepan. They measured 10 mm in diameter and ≈1.5 mm in height, and were loaded in the longitudinal direction with an acceleration of 1 mm min^−1^. The data for the calculation of the compressive modulus were continuously recorded at a frequency of 50 Hz using the Emperor XT Force software. To determine the compressive modulus, forces of 0.25, 0.5, 1, and 2.5 N, respectively, were applied to the samples and kept for 60 s. Subsequently, an unloading period (0 N) followed for 300 s. The resulting force‐displacement‐time data were used to calculate the compressive modulus (*E*_C_) of the material and the tensile modulus (*E*_T_).^69^

*Determination of the Mechanical Properties: Tensile Testing*: For the tensile experiments standardized 3–4 mm thick stripes, measuring 30 mm in length and 12 mm in width, were cut out from the sample material. These stripes were loaded in the longitudinal direction with an acceleration of 5 mm min^−1^ until tearing off. The continuously recorded force, displacement and time data (sampling rate: 50 Hz) were used to calculate the UTS, the ME, as well as the tensile modulus (*E*_T_).^69^ The same procedure was applied also for bovine muscle samples from the lower leg; the muscle was obtained from a local butcher. The samples were cut to the same dimensions as the KG/PVA materials and analyzed as described above.

*Cultivation of Human Mesenchymal Stem Cells*: Human MSCs, obtained from bone marrow of healthy nondiabetic adult volunteers, were purchased from Lonza Cologne (Cologne; Germany). They were cultivated as described before.^17^ Shortly, the cells were maintained in 75 cm^2^ flasks and cultivated in DMEM medium, supplemented with 10% FCS (Biochrom, Berlin; Germany) and 0.5 mg mL^−1^ of gentamycin, 100 units mL^−1^ of penicillin, 100 µg mL^−1^ of streptomycin, and 1 × 10^−3^ M pyruvate (#P2256 Sigma‐Aldrich). The assays were incubated in a humidified incubator at 37 °C.

For plating of the MSCs onto the KG/PVA cryogel, the material was prepared first in 6‐well plates (#M8562; Sigma). A final volume of 1 mL of “KG2/PVA‐cryogel” was prepared in each well following the described procedure by freezing‐thawing, followed by incubation with 5% CaCl_2_. For the preparation of the “KG2/PVA/polyP:NP‐cryogel” in the 6‐well plates the samples were treated with Na‐polyP and finally with 5% CaCl_2_. After extensive washing with medium, 3 mL cell suspension (20 × 10^3^ cells mL^−1^) were placed onto the cryogel prepared onto the bottom of the well plates. For the determination of the attachment properties of the MSC the incubation was performed for 8 or 24 h. In one series of experiments, the samples were inspected by low‐resolution SEM and after fixation with paraformaldehyde and glutaraldehyde.^9^ The samples were coated with gold; in a second one the cryogels were exposed to Calcein AM (#17783; Sigma) to analyze the cells^70^ within the cryogel by cLSM.^71^

*Cell Proliferation/Viability Assay*: MSCs were seeded into the 6‐well plates at a density of 10^4^ cells per well and were cultured in DMEM medium/10% FCS for the indicated period of time (total volume of 3 mL). The parallel assays were set. First, in the control wells no further material/cryogel was added. In the second and third series, the polyP‐free cryogels (height of the cushion: 1 mm) were prepared using the “KG2/PVA‐cryogel” composition and for the polyP‐containing cushions the “KG2/PVA/polyP:NP‐cryogel” formula was applied. The cells were layered onto the gels.

The proliferation/viability assays were performed using the XTT Cell Proliferation Kit II [2,3‐bis‐(2‐methoxy‐4‐nitro‐5‐sulfophenyl)‐2*H*‐tetrazolium‐5‐carboxanilide], purchased from Roche (Mannheim; Germany), as outlined.^72^ The cell growth/metabolic activity of the living cells was determined on the basis of the extent of oxidation of the tetrazolium salt. The absorbance was determined at 450 nm and the values were subtracted by the background values (500 nm). The cells were incubated for up to 7 days. Ten parallel experiments were performed.

*Preparation of Karaya Gum/Polyvinyl Alcohol Microspheres*: The microspheres were prepared as follows. The aqueous phase was composed with 10 g of PVA in 100 mL of distilled water and heated to 90 °C (while stirring for 3 h) to dissolve the polymer. In parallel, 10 g of KG were dissolved in 100 mL of water at 90 °C for 3 h. Then, 50 mL of both solutions were mixed together on a magnetic stirrer. The oil phase contained in 200 mL of paraffin oil (#18512, Sigma) 10% (wt/wt) Tween‐80 (#P4780, Sigma). To prepare the microspheres 20 mL of the aqueous phase were added to the oil phase under stirring (1500 rpm; room temperature) during a 30 min period. After that, 10 mL of a CaCl_2_ solution (from the stock solution of 1 g CaCl_2_/10 mL water) were filled into a syringe, and in separate but at the same time, 10 mL of a Na‐polyP solution (stock solution of 0.2 g/10 mL water) were likewise filled into a syringe. Then, in parallel, these two solutions (both 10 mL) were added under stirring, drop‐by‐drop, to the oil phase. After stirring for 3 h at 500 rpm the suspension was taken. The particles were sedimented during 10 min. The oil phase was removed by decantation. The sediment was washed three times in distilled water. Finally the material was centrifuged at 1000×g and the spheres were freeze‐dried at −80 °C; they are termed “KG/PVA/polyP:MS”. The polyP‐free microspheres were prepared in the same way without adding polyP; “KG/PVA:MS”. The “KG/PVA/polyP:MS” samples contained ≈8.5 wt% polyP, as determined by EDX.

*Animal Studies*: The biocompatibility studies were performed with Wistar rats (male gender; age of two months) weighting between 250 and 300 g.^73^ All experimental procedures were approved by the ethics committee at the Dongzhimen Hospital at the Beijing University of Chinese Medicine (No. 5 Haiyuncang Road, Dongcheng District, Beijing 100700; Beijing Committee of Science and Technology). The certificate number for the approval is 2012‐0001a; the experimental studies were performed by Dr. Xing YU. Four animals per each group were used.

The animals were kept at constant room temperature (22 ± 2 °C), controlled 12 h light/dark cycle and humidity (≈50%); diet and water were provided ad libitum. As described,^73^ preoperatively the animals were treated with Ciprofloxacin (Bayer, Leverkusen; Germany) at a dose of 10 mL kg^−1^ of body weight for antibiotic prophylaxis. Subsequently, the animals were narcotized with chlorpromazine (Smith, Kline & French, Philadelphia; PA)/Ketamine (Ketanest; Pfizer, Groton; CT) using the intramuscular route. After routine disinfection, incisions of ≈1 cm were made in the right and left half and oriented perpendicularly to the vertebral axis at the upper limb level. After skin incision, the muscle was incised and dissected to allow the insertion of the microspheres. The implanted material (≈20 mg in a volume of 100 mL) was introduced into the muscle and stabilized there in the deeper layer.^74^, ^75^, ^76^ After a period of 2 or 4 weeks the animals were sacrificed and the specimens with the surrounding tissue were dissected and sliced. The samples were inspected macroscopically for inflammation, infection and discoloration.

Two groups of studies were performed; one group received the microspheres without polyP “KG/PVA:MS”, and one group with polyP “KG/PVA/polyP:MS”.

For histological inspection the samples were fixed in formalin, sliced, stained with Mayer's hematoxylin (#MHS1; Sigma) and then analyzed by optical microscopy (using an Olympus AHBT3 microscope).^77^

*Microscopic Analysis–Pore Size Determinations*: The high‐resolution SEM images were obtained with a Zeiss Gemini 1530 (Zeiss Oberkochem; Germany). For low‐resolution SEM studies an ESEM XL‐30 machine (Philips, Eindhoven; Netherlands) was applied.^78^ cLSM was performed with a Leica apparatus (Microsystems, Bensheim; Germany) of Calcein AM‐stained cells. Serial confocal images (64 z‐slices) were captured and the 3D overlaid image stacks were computed using an average projection algorithm provided with the TCS‐SP software. The settings of the microscope have been given recently.^79^

Pore‐size determinations were performed with SEM/ESEM XL images obtained from gold sputtered cryogel samples, as described.^80^ Then ImageJ analysis software was applied to determine the averaged mean pore sizes; randomly selected samples have been analyzed.

*Statistical Analysis*: The values reported are the average ± standard deviations. Statistical analyses were performed with the one‐way ANOVA test, by using SigmaStat 3.5 software (Dundas Software Ltd, Toronto; Canada). Values of *p* < 0.05 were considered statistically significant.

## Conflict of Interest

The authors declare no conflict of interest.
